# Recalcitrant carbohydrates after enzymatic hydrolysis of pretreated lignocellulosic biomass

**DOI:** 10.1186/s13068-016-0629-4

**Published:** 2016-10-06

**Authors:** María Ángeles Bermúdez Alcántara, Justyna Dobruchowska, Parastoo Azadi, Bruno Díez García, Fernando P. Molina-Heredia, Francisco Manuel Reyes-Sosa

**Affiliations:** 1 Department of Biotechnology, Abengoa Research, Campus Palmas Altas, C/Energía Solar no 1, 41014 Seville, Spain; 2 Complex Carbohydrate Research Center, University of Georgia, Athens, 30602 Georgia; 3 Instituto de Bioquímica Vegetal y Fotosíntesis, Universidad de Sevilla y CSIC, Américo Vespucio 49, 41092 Seville, Spain

**Keywords:** Enzymatic hydrolysis, Lignocellulosic biomass, Recalcitrant carbohydrates, Bioethanol

## Abstract

**Background:**

To reduce the cost of the enzymes for the hydrolysis of lignocellulosic biomass, two main strategies have been followed: one, the reduction of enzyme dosing by the use of more efficient and stable enzymatic cocktails; another, to include accessory enzymes in the cocktails to increase yields by reducing the recalcitrant carbohydrate fraction remaining at the end of the process. To guide this second strategy, we have explored the chemical bond composition of different fractions of recalcitrant carbohydrates after enzymatic hydrolysis.

**Results:**

Two lignocellulosic feedstocks of relevance for the biofuels industry have been analyzed, corn stover and sugarcane straw. On comparing the composition of chemical bonds of the starting pretreated material with samples after standard and forced hydrolysis (with enzyme overdosing), we obtained similar sugar and chemical bond composition.

**Conclusions:**

This suggests that the current enzymatic cocktails bear the set of enzymes needed to hydrolyze these feedstocks. From our point of view, the results show the need for a parallel fine-tuning of the enzymatic cocktails with the pretreatment process to maximize sugar release yield.

## Background

The interest of biochemical deconstruction of lignocellulosic biomass into sugars for the production of ethanol fuel or chemicals has been increasing in the last decades due to oil prices volatility, together with the lower carbon emissions profile of the biochemical route with direct benefit in the mitigation of global warming and climate change. Lignocellulosic materials are a renewable and abundant source of carbon for the production of fuels and chemicals. They can be obtained from low-cost resources like agricultural and forest residues, municipal solid waste, waste paper and energy crops. These materials contain polymeric sugars that can be hydrolyzed and subsequently fermented to ethanol by microorganisms [1].

Lignocellulosic material is composed mainly of three polymers: cellulose, hemicellulose and lignin, which are associated in a complex matrix with different composition depending on the type, species and source of the biomass [2]. The presence of these complex interlinked polymers presents a physical barrier to protect cellulose from degradation. This compact and rigid structure is the cause of biomass recalcitrance to hydrolysis and deconstruction. The factors that contribute to biomass recalcitrance include: crystallinity and degree of polymerization of cellulose; accessible surface area (or porosity); protection of cellulose by lignin; cellulose sheathing by hemicellulose; and fiber strength [3, 4].

While the enzymatic breakdown of lignocellulosic biomass is a complicated process, involving many activities which work in tandem to decompose a heterogeneous and naturally recalcitrant substrate, the understanding of both known and yet-to be discovered enzymes and activities has increased significantly in recent years [5].

To increase the yield of enzymatic hydrolysis at the industrial scale, a physico-chemical pretreatment of the biomass is required, whose main objective is altering the chemical composition and physical structures of biomass to remove the recalcitrant barriers and enhance the enzymatic digestibility of cellulose, to allow the exposed complex carbohydrates such as cellulose and hemicelluloses to be readily hydrolyzed to fermentable sugars.

There are many comprehensive reviews where different pretreatment technologies based on physical, chemical and biological methods have been described [2, 3, 6]. Basically, an effective pretreatment method should be cheap (both in capital and operating costs), effective on a wide range of lignocellulosic materials, require minimum preparation/handling steps prior to pretreatment, ensure recovery of all of the lignocellulosic components in a useable form, and provide a cellulosic stream that can be efficiently hydrolyzed with low concentrations of enzymes [2].

Due to the interplay of the efficiency of the pretreatment with the enzymatic hydrolysis yields, it is often difficult to ascertain which of the two processes limits the overall yield. One possible limitation could be that the enzyme mixture would lack specific activities to unlock key chemical bonds in the polymers, thus limiting the overall sugar yield. An alternative hypothesis would be that the cocktail would be adequately formulated, but a portion of the biomass could be inefficiently pretreated, provoking a limitation on enzymatic hydrolysis yield. To test these hypothesis, in the present study we characterized two different lignocellulosic materials, corn stover and sugarcane straw, both pretreated by diluted acid/steam explosion, following hydrolysis under low and high enzyme loading. After the hydrolysis, the chemical composition of the recalcitrant materials was analyzed to unveil the composition of these recalcitrant materials using several approaches such as solid-state nuclear magnetic resonance (NMR) spectroscopy, glycosyl composition determined by using gas chromatography/mass spectrometry (GC/MS) of the per-*O*-trimethylsilyl (TMS) derivatives, and per-O-methylation linkage analysis of glycosyl residues to have a global view of the chemical linkages present in the material as compared to the starting pretreated material. No different chemical bond composition was found when starting and recalcitrant materials were compared. This supports the hypothesis that pretreatment rather than enzymatic cocktail reformulation represents the main bottleneck in sugar release yields.

## Methods

### Corn stover and sugarcane straw

Pretreated corn stover and sugarcane straw (from now on, PCS and PSCS, respectively) were obtained from Abengoa Bioenergy Biomass Pilot Plant in York, Nebraska, USA. A 1-inch hammer mill screen was used to grind the material. Pretreatment of the milled material was performed by steam explosion using 2.3 % (w/v) sulfuric acid for spray impregnation of the biomass in a continuous digester, previous to pressurizing with steam at 150 psig for 2 min; a final dry matter content of 37.4 and 43.4 % was achieved, respectively, for PCS and PSCS. The compositional analysis of the biomass was according to the standard biomass analytical procedures by NREL [7].

### Enzymatic hydrolysis

Hydrolysis of the pretreated biomass (20 g) was performed in 100 mL borosilicate glass bottles with airtight screw caps. Water was added to adjust the solid loading to 20 % of water insoluble solids (WIS; based on substrate). The pH was initially adjusted to 5.5 by addition of aqueous NH_4_OH. The enzyme loading was 10 and 100 mg protein per gram glucan of C1 enzyme preparation (supplied by Abengoa). The hydrolysis was incubated at 50 °C with orbital shaking at 150 rpm for 72 h. Samples were taken at *t* = 0 and *t* = 72 h of hydrolysis and were processed for analysis according to Kristensen et al. [8] due to the higher density of the hydrolysate at 20 % of WIS. The analytes were quantified in weight/weight (g/kg).

### Carbohydrate analysis

After enzymatic hydrolysis, samples were filtered and analyzed by high-performance liquid chromatography (HPLC) using an Aminex HPX-87H 300 mm × 7.8 mm column with 9 µm particle size (Bio-Rad, California, USA). The analyses were performed at 60 °C under isocratic conditions with 5 mM H_2_SO_4_ as mobile phase at a flow rate of 0.6 mL/min with 20 µL injection volume. Carbohydrates (glucose, xylose and arabinose) were analyzed using a refractive index detector.

### Sample preparation

After hydrolysis, the enzymes were inactivated by boiling for 15 min at 100 °C. The samples were centrifuged (16,000×*g*, 30 min, 4 °C) and the pellets washed with Milli-Q water at 50 °C for 1 h, repeating the washing until no soluble sugars were detected by HPLC in the supernatants. Then, recalcitrant materials were freeze dried. Dry samples were milled in a hammer mill, passed through a 250 µm sieve and their humidity content determined using a Shimadzu (Kyoto, Japan) heating balance. All the component contents are expressed on dry weight basis as average with a standard deviation of duplicate determinations for each sample.

### Glycosyl composition by GC/MS of TMS derivatives of methyl glycosides

Glycosyl composition analysis was performed by combined GC/MS of the per-*O*-trimethylsilyl derivatives of the monosaccharide methyl glycosides produced from the sample by acidic methanolysis. 400 µg samples were placed in a screw-cap tube with 20 µg of inositol as internal standard and hydrolyzed with 2 M trifluoroacetic acid (TFA) at 120 °C for 1 h. The hydrolysis products were dried by adding iso-propanol and methanol to remove TFA. Methyl glycosides were then prepared from the freeze-dried sample following the mild acid treatment by methanolysis in 1 M HCl in methanol at 80 °C (16 h), followed by re–*N*-acetylation with pyridine and acetic anhydride in methanol (for detection of amino sugars). The sample was then *O*-trimethylsilylated by treatment with Tri-Sil at 80 °C (0.5 h). These procedures were carried out as previously described by Merkle and Poppe [9]. GC/MS analysis of the TMS methyl glycosides was performed on an Agilent 7890A GC interfaced to a 5975C MSD, using a Supelco EC-1 fused silica capillary column (30 m × 0.25 mm ID).

### Per-*O*-methylation and linkage analysis of neutral sugars

For glycosyl linkage analysis, the sample was permethylated, depolymerized, reduced, and acetylated. The resulting partially methylated alditol acetates (PMAAs) were analyzed by GC/MS as described by York et al. [10]. Initially, dry samples were suspended in about 300 µL of dimethyl sulfoxide and placed on a magnetic stirrer for 1 week. The samples were then permethylated by the method of Ciucanu and Kerek (treatment with sodium hydroxide and methyl iodide in dry DMSO) [11]. Each sample was incubated with NaOH for 15 min, then methyl iodide was added and left for 45 min. The base was then added for 10 min and finally more methyl iodide was added for 40 min. This addition of more methyl iodide and NaOH base was to ensure complete methylation of the polymer. Following the sample workup, the permethylated material (PMAA) was hydrolyzed using 2 M TFA (2 h in sealed tube at 121°C), reduced with NaBD_4_, and acetylated using acetic anhydride/TFA. The resulting PMAAs were analyzed on a Hewlett Packard 7890A GC interfaced to a 5975C MSD mass selective detector, electron impact ionization mode (EI-MS); separation was performed on a 30 m Supelco 2380 bonded phase fused silica capillary column.

### Solid-state NMR

Solid-state ^13^C NMR experiments were carried out in a Bruker Avance III 600 WB with a magnetic field of 17.09 T and equipped with a 4 mm multinuclear probe MAS NMR. ^13^C resonance frequency in this magnetic field is 150.9 MHz.

NMR experiments were performed on the untreated sample and also on the solid biomass fractions resulting from enzymatic hydrolysis samples were packed in zirconium oxide rotors and were spun at 12 kHz.

The proton decoupled ^13^C NMR experiments were carried out using a pulse of 30º of 1.37 µs for ^13^C, a relaxation time of 10 s, acquisition time of 0.031 s and a scan number of 4800, corresponding to 11 h and 10 min for each sample. The chemical shifts are expressed in ppm and they were referenced with TMS (δ = 0 ppm).

Assignments of peaks are described in Table 1 based on previous NMR analysis on literature [12, 13].

**Table 1:** Assignment of NMR peaks 1–17 indicated on the spectrum in Fig. 3

Peak number	Chemical group	^13^C chemical shift (ppm)
1	Aliphatic lignin carbons not bound to oxygen	32.5
2	Aryl methoxyl carbons of lignin	56.2
3	C6 carbon of non-crystalline/amorphous cellulose, C6 carbon of hemicelluloses, OCγH2 carbons of lignin	62.5
4	C6 carbon of crystalline cellulose	64.8
5	C2,3,5 of cellulose, OCαH2 carbons of lignin	72.5
6	C2,3,5 of cellulose and hemicelluloses	74.4
7	C4 carbon of non-crystalline cellulose and hemicelluloses, OCβH2 carbons of lignin	83.5
8	C4 carbon of crystalline cellulose	87.9
9	C1 carbon of hemicelluloses	101.8
10	C1 carbon of cellulose	105.0
11	C2 and C6 aromatic carbons of syringyl and C5 and C6 aromatic carbons of guaiacyl in lignin	110.0–115.0
12	C2 of aromatic carbons guaiacyl in lignin	126.6
13	C1 and C4 aromatic carbons of syringyl^a^	136.9
14	C3 and C5 aromatic carbons of syringyl^a^ and C1 and C4 aromatic carbons of guaiacyl in lignin	148.0
15	C3 and C5 aromatic carbons of syringyl^b^ in lignin	153.5
16	Carboxyl groups of lignin	163.0–180.3
17	Carboxyl groups of hemicelluloses	173.6

These assignments were based on published material

^a^ Non-etherified arylglycerol β-aryl ethers

^b^ Etherified arylglycerol β-aryl ethers

## Results and discussion

### Composition of pretreated corn stover and pretreated sugarcane straw samples

The compositional analysis of each material was carried out as described in materials and method. Table 2 shows carbohydrates (cellulose and hemicellulose components) and lignin composition of both materials. The insoluble part of each material showed some similarities relating to insoluble sugar content, although pretreated sugarcane straw exhibited more xylan content than pretreated corn stover, probably due to a lower effect of the pretreatment. A small percentage of mannan is present in pretreated sugarcane straw, but is not detected in pretreated corn stover.

**Table 2:** Chemical composition of the insoluble pretreated corn stover and pretreated sugarcane straw samples

Structural component	Insoluble biomass composition (% DM)	
	Pretreated corn stover	Pretreated sugarcane straw
Cellulose		
Glucan	34.42 ± 0.21	34.52 ± 0.32
Hemicellulose		
Xylan	3.26 ± 0.36	4.37 ± 0.40
Arabinan	0.33 ± 0.04	0.55 ± 0.07
Mannan	0.00 ± 0.00	0.24 ± 0.03
Klason lignin	18.03 ± 0.08	18.51 ± 0.05
Ash	2.40 ± 0.10	2.41 ± 0.08
Total	58.44 ± 0.79	60.59 ± 0.95

Contents are expressed on a dry weight basis as an average (±standard deviation) of duplicate determinations

Samples were pretreated using the two-stage acid hydrolysis method as described in experimental procedures

### Enzymatic hydrolysis of pretreated corn stover and pretreated sugarcane straw

**Fig. 1:**
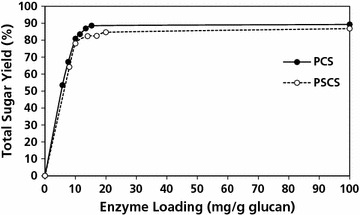
Sugar release as a function of enzyme dose. Total free sugar yield was determined after 72 h of enzymatic hydrolysis at different enzyme loading of C1. The sample were analyzed in duplicate (values are mean ± SD)

Samples were hydrolyzed using a cellulolytic cocktail produced by C1 strain of *Myceliophthora thermophila* [14]. This strain developed by Abengoa is able to achieve more than 100 g/L of total extracellular protein at the industrial-scale fermenters. More than 90 % of the extracellular protein produced consists of a mixture of cellulases, of which 40–55 % are cellobiohydrolases, 20–25 % are endoglucanases among others betaglucosidases, betaxilosidases, polysaccharide monooxygenases, xylanases and xyloglucanases, arabinofuranosidases, acetylxylan esterases, and alfa and betagalactosidases. Due to its lower ethanol cost contribution, this enzymatic cocktail produced is preferred instead of others from well-known fungi like *Aspergillus* sp. or *Trichoderma reesei* for recently developed biorefineries. Figure 1 shows the total sugar yield achieved for the two pretreated substrates in response to increases in enzyme dose. For both materials, assays were done at 20 % total solids at 50 °C for 72 h. Above 50 mg of enzyme per gram of glucan, the sugar yield reached 90 % and did not increase further, leaving about 10 % of potential sugar unreleased.

This gap between theoretic potential sugars and real yield at high enzyme loading might be explained by two hypothesis: either pretreatment of the material is not enough to recover all potential sugars present in the biomass, or the structure of the recalcitrant material is complex and new or different accessory enzymes are needed to release all C5 or C6 monomeric sugars. To elucidate this, different approaches were undertaken using glycosyl analysis of the recalcitrant materials.

### Glycosyl composition analysis

Glycosyl composition analysis of the recalcitrant material was performed by combined GC/MS of the per-*O*-trimethylsilyl derivatives of the monosaccharide methyl glycosides produced from the samples by acidic methanolysis. The data are presented in Table 3.

**Table 3:** Glycosyl residue content

Dosage (mg/g)	Glycosyl residue content (mol %; Min–max)					
	PCS			PSCS		
	0	10	100	0	10	100
Residue						
Glucose	31.7–33.3	32.4–36.1	26.4–26.6	16.9–18.2	38.7–40.7	28.6–30.6
Xylose	51.1–53.4	51.1–53.4	53.6–54.8	64.8–66.9	45.4–47.0	46.5–50.7
Arabinose	13.0–13.1	11.6–13.2	15.8–16.6	14.8–15.4	12.1–12.4	14.0–14.7
Mannose	0.5–0.7	1.0–1.2	2.9–3.4	0.8–1.0	1.8–1.9	6.7–8.1
Galactose	1.3–1.9	nd	nd	0.6–0.7	nd–0.4	nd
Total monosaccharides (mg/g)	221.6–301.1	37.1–71.6	21.4–31.4	250.9–653.5	51.4–51.8	25.7–39.4

Ribose, rhamnose, fucose, glucuronic acid, galacturonic acid, *N*-acetyl galactosamine, *N*-acetyl glucosamine and *N*-acetyl mannosamine were not detected in any material

Glycosyl residue content (mol %) of each sample at initial time (dosage 0 mg/g), and the insoluble part after enzymatic hydrolysis at lower dosage (10 mg/g) and higher dosage (100 mg/g) loading of C1 enzyme related to total dry biomass

*nd* not detected

Only five different monosaccharides were detected using this analytical technique (arabinose, glucose, xylose, mannose and galactose), and those monomers that were more represented in both materials were glucose and xylose, matching with the previous compositional analysis (Table 2). No other saccharides such as ribose, rhamnose, fucose, glucuronic acid, galacturonic acid, *N*-acetyl galactosamine, *N*-acetyl glucosamine, and *N*-acetyl mannosamine were detected in any material. Galactose was only detected in the starting materials at low concentration, but not in the insoluble portion after enzymatic hydrolysis. As expected, at higher enzyme dosage, the glucose content present in recalcitrant material as cellulose and hemicellulose diminished compared to low dosages (Table 3). Comparing different enzyme dosages with non-hydrolyzed material monosaccharides proportions present in the recalcitrant material of corn stover remains almost constant. PSCS shows the highest xylose percentage, but it is reduced enzymatically to similar percentages than obtained with PCS. Mannose level was almost constant at low dosage; however, at the high dosage, this sugar became more abundant in both recalcitrant materials. This fact might indicate that mannose links in the recalcitrant material are not released by the enzymatic cocktail. To identify the different chemical bonds linking all these carbohydrates, a glycosyl linkage analysis was performed.

**Fig. 2:**
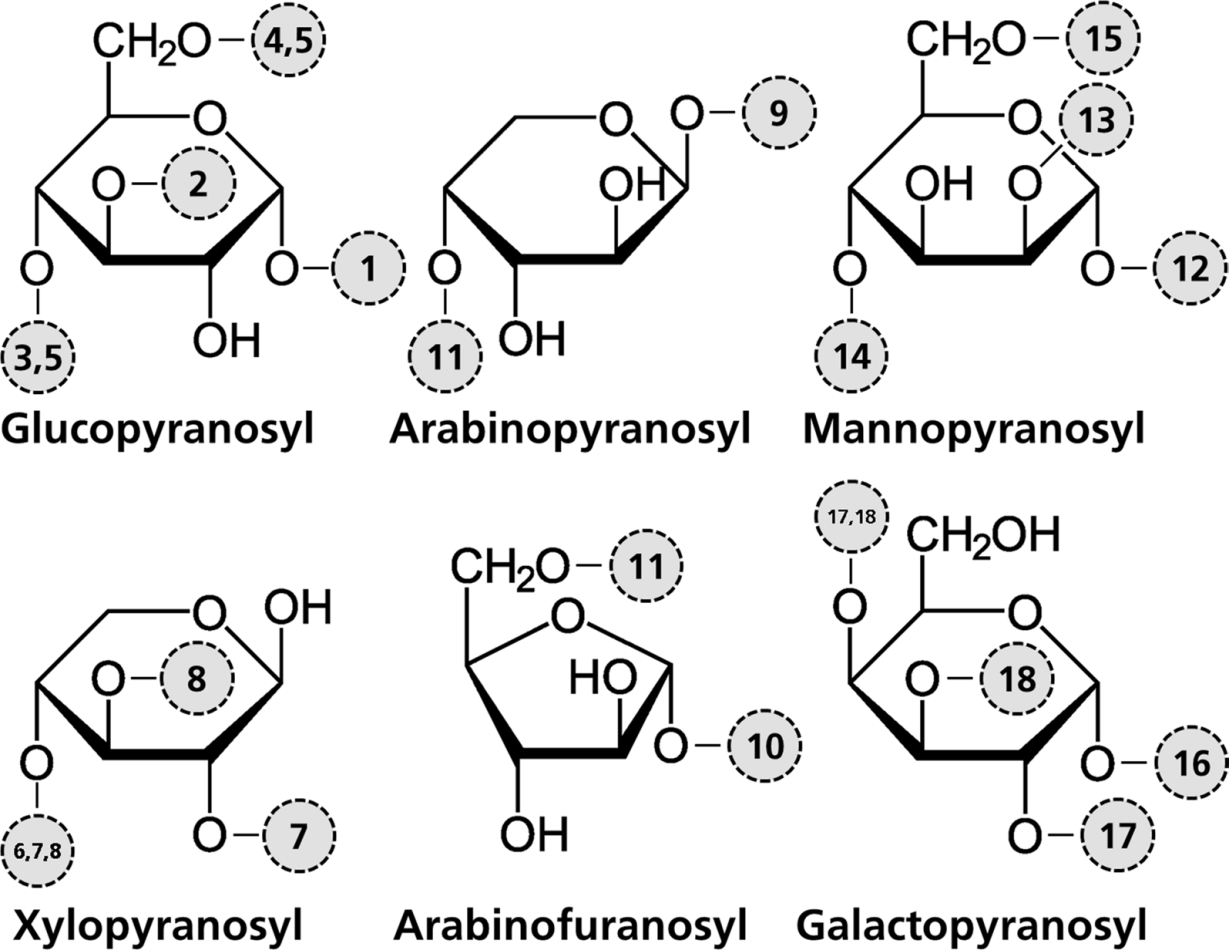
Schematic structure of linkage type on model compounds. Glucopyranosyl residues: terminally linked (*1*), 3 linked (*2*), 4 linked (*3*), 6 linked (*4*), 4,6 linked (*5*); Xylopyranosyl residues: 4 linked (*6*), 2,4 linked (*7*), 3,4 linked (*8*); arabino(pyra/fura) nosyl residues: terminally linked pyranosyl (*9*), terminally linked furanosyl (*10*), 4 linked pyranosyl or 5 linked furanosyl (*11*); mannopyranosyl residues: terminally linked (*12*), 2 linked (*13*), 4 linked (*14*), 6 linked (*15*); galactopyranosyl residues: terminally linked (*16*), 2,4 linked (*17*), 3,4 linked (*18*)

For glycosyl linkage analysis, per-*O*-methylation and linkage analysis of neutral sugars was carried out. The sample was permethylated, depolymerized, reduced, and acetylated; and the resulting PMAAs analyzed by GC/MS. Linkage types detected using this technique were those corresponding to the five monosaccharides formerly determined by TMS analysis, showing no other sugars in the biomass composition (Table 4). A schematic diagram of each glycosyl linkage type is shown in the Fig. 2.

**Table 4:** Glycosyl linkage content

Residue linkage type	Dosage (mg/g)	Glycosyl linkage content (%)					
		PCS			PSCS		
		0	10	100	0	10	100
Glucopyranosyl	Terminally linked (1)	5.4	3.5	6.2	7.2	9.4	7.1
	3 linked (2)	1.9	0.4	0.5	1.2	0.5	0.6
	4 linked (3)	63.3	72.1	62.8	71.6	67.8	52.7
	6 linked (4)	0.0	0.3	0.4	0.0	0.5	0.9
	4,6 linked (5)	1.6	1.8	1.7	1.5	1.8	2.3
Xylopyranosyl	4 linked (6)	13.6	11.3	13.4	8.0	8.7	13.4
	2,4 linked (7)	0.0	0.4	0.3	0.0	0.3	0.0
	3,4 linked (8)	0.9	1.3	2.0	0	1.3	3.6
Arabino(pyra/fyra)nosyl (Ara*p*/Ara*f*)^a^	Terminally linked (9)	4.8	1.2	1.8	2.8	1.6	2.9
	Terminally linked (10)	0.9	2.0	1.7	0.4	1.7	5.3
	4 linked Ara*p* or 5 linked Ara*f* (11)^a^	1.0	0.0	1.9	0.0	1.9	5.6
Mannopyranosyl	Terminally linked (12)	0.7	0.1	1.1	0.7	0.6	1.4
	2 linked (13)	0.0	0.0	0.7	0.0	0.0	0.9
	4 linked (14)	2.7	1.1	0.5	3.4	1.0	0.0
	6 linked (15)	0.0	0.0	0.1	0.0	0.0	0.0
Galactopyranosyl	Terminally linked (16)	0.9	1.0	0.6	0.9	0.3	0.0
	2,4 linked (17)	0.6	0.8	1.7	0.6	0.8	0.5
	3,4 linked (18)	1.4	1.7	1.6	1.4	1.1	1.7

^a^ 4-linked Ara*p* and 5-linked Ara*f* give rise to the same PMAA and can thus not be distinguished by this method. *Numbers* within parentheses are related with the drawings in Fig. 2

Glycosyl linkage residue of each sample at initial time (dosage 0 mg/g), and the insoluble part after enzymatic hydrolysis at lower dosage (10 mg/g) and higher dosage (100 mg/g) loading of C1 enzyme related to total dry biomass. Samples were performed in duplicate

According to per-*O*-methylation and linkage analysis, 4 linked glucopyranosyl residue is the major component of all samples and a significant amount of 4 linked xylopyranosyl residues is also present in all samples. The possible origin of the linked residues detected is presented in Table 5.

**Table 5:** Possible origin of the detected linked residues

Residue	Linkage type	Model compound^a^	Putative enzyme involved
Glucopyranosyl	Terminally linked (1)	Pullulan	Eg/Cbh/Bgl
	3 linked (2)	Lichenan	Ace/Fe
	4 linked (3)	Glucan	Eg/Cbh/Bgl
	6 linked (4)	Pullulan, stachyose	Ace/Fe/Aabn
	4,6 linked (5)	Xyloglucan	Eg/Cbh/Bgl
Xylopyranosyl	4 linked (6)	Xylan	Xyl/Bxl
	2,4 linked and 3,4 linked (7, 8)	Arabinoxylan	Abn/Xyl/Bxl
Arabino(pyra/fyra)nosyl (Ara*p*/Ara*f*)	Terminally linked (9, 10)	Arabinose	Abn
	4-linked Ara*p* or 5-linked Ara*f* (11)	Debranched arabinan	Aabn
Mannopyranosyl	Terminally linked (12)	Mannose	Bman
	2 linked and 6 linked (13, 15)	Acetylation^b^	Ace/Fe
	4 linked (14)	Mannan	Bman
Galactopyranosyl	Terminally linked (16)	Galactose	Bgal
	2,4 linked and 3,4 linked (17, 18)	Galactan^b^	AGal/Bgal

^a^ Based on previously published data

^b^ Proposed hypothetical model compound

*Numbers* within parentheses are related to drawings in Fig. 2

*Abn* arabinofuranosidases, *Aabn* alfaarabinofuranosidases, *Xyl* xylanases, *Bxl* betaxylosidases, *Eg* endoglucanases, *Cbh* cellobiohydrolases, *Bgl* betaglucosidases, *Ace* acetylxylan esterases, *Fe* feruroyl esterases, *Bman* betamannosidases, *Agal* alfagalactosidases, *Bgal* betagalactosidases

The linkage information is deduced from the knowledge of model compounds

The linkage information is based on published identification with model compounds [15]. Apart from the predominant 4 linked glucopyranosyl residues mentioned, four additional types of glucose linkages were detected. Among them, 6-linked glucopyranosyl residues that were not detected in the initial material were present in a small proportion after enzymatic hydrolysis regardless of the dosages. This residue might be released by the action of different enzymes, like, depending on the modifications present, acetylxylan–feruloyl esterases or α-arabinofuranosidases.

The 3-linked glucopyranosyl residues became less represented after enzymatic hydrolysis compared to the initial material and remained almost constant at lower and higher enzyme loading.

In the case of xylose residues, 3,4-linked xylopyranosyl residues increased their proportion after hydrolysis with higher loading on the enzyme. These kinds of links are usually found in arabinoxylan structures; consequently, enzymes that could be involved would be arabinofuranosidase, xylanase or beta-xylosidase, among others.

On the other hand, arabinopyranosyl, galactopyranosyl, and mannopyranosyl residues remained more or less constant in all conditions and were less abundant.

It has to be noted that quantitative composition analysis data are inconsistent with the linkage analysis data. The linkage analysis indicated higher amounts of 4-linked glucopyranosyl residues in all samples, while composition analysis suggested lesser amount of glucose. These results can be explained by the fact that the permethylation step in linkage analysis allowed to solubilize cellulose resulting in more efficient hydrolysis.

All these analyses revealed that most linkages belonged to glucan and xylan, specifically, beta-1,4-glucan and beta-1,4-xylan. Therefore, although there are some residues from other carbohydrates, the main recalcitrant material would be composed of these two polymers. Enzymes that are involved in their breakdown are endoglucanases, cellobiohydrolases, and beta-glucosidases in the case of glucan, and xylanase and beta-xylosidase, in the case of xylan, enzymes that are already present in the C1 enzymatic cocktail preparation. The same results were also obtained repeating the same analysis with other available commercial cellulolytic cocktail like Ctec3 from Novozymes (data not shown).

Because the enzymes required to hydrolyze the types of linkages found are abundant in the enzyme cocktails used and, moreover, they have been able to solubilize most of the material in the prehydrolysis phase, we could deduce that the recalcitrant material remaining is not accessible to the enzymes and, hence, it cannot be hydrolyzed. These pockets of inaccessible recalcitrant polysaccharides could come from an incomplete pretreatment of the starting biomass.

### Solid-state ^13^C nuclear magnetic resonance

Further information on chemical composition for recalcitrant samples was obtained by high-resolution ^13^C solid-state nuclear magnetic resonance. For organic matter applications, one of the most quantitative ^13^C NMR techniques is probably the DPMAS method, consisting of the excitation of the ^13^C nuclei by a single π/2 pulse, followed by acquisition under ^1^H decoupling and fast magic-angle spinning. This method can readily provide information on modifications taking place in specific chemical groups, without great experimental effort concerning sample preparation and analysis, which makes it fast [16, 17].

**Fig. 3:**
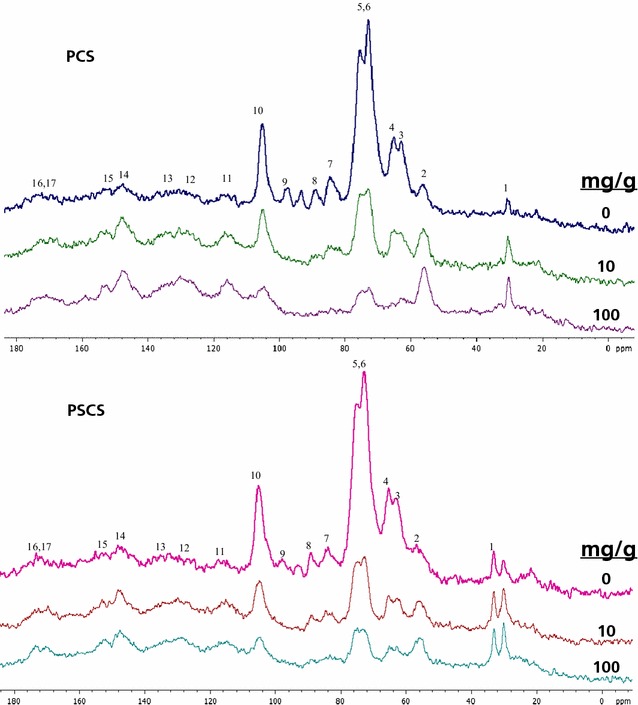
Identification of 13 signals in non-hydrolyzed and hydrolyzed materials. Solid stage ^13^C high-power proton decoupling magic-angle spinning (^13^C HPDC/MAS) nuclear magnetic resonance (NMR) spectra of pretreated corn stover (up; PCS) and pretreated sugarcane straw (down; PSCS) at several enzyme loadings before enzymatic hydrolysis (0 mg protein/g glucan) and after enzymatic hydrolysis (using 10 and 100 mg protein/g glucan)

Figure 3 shows the ^13^C NMR spectrum for the PCS samples at different enzyme loadings. This spectrum is very similar to those described in the literature [18–20].

The cellulose region (60–110 ppm) is typical of pretreated corn stover samples. Signals 3 and 7 (at 63 and 84 ppm) were assigned, respectively, to C6 and C4 carbon from amorphous cellulose. On the other hand, signal 4 and 8 (at 65 and 88 ppm) were assigned to C6 and C4 carbon in crystalline cellulose. It is difficult to clearly differentiate the signals of lignin, hemicellulose, and cellulose because there is a contribution of all bound carbons along the entire spectral region. However, contribution of hemicellulose and lignin is smaller in the region from 60 to 110 ppm. Lignin signal becomes more important from 110 to 180 ppm, while hemicellulose signals contribute to peaks 3, 6, 7, 8, 9, and 17.

Comparing the spectra at the two enzyme loadings, the same peaks from cellulose signals (3, 4, 5, 6, 7, 8, and 10) were observed, indicating that there was still a fraction of cellulose present after enzymatic hydrolysis at the higher enzyme loading (Fig. 3). This would support the hypothesis obtained with the glycosyl analysis about the presence of cellulose and hemicellulose rests blocked to enzyme action. The same results were observed for both PCS and PSCS materials.

## Conclusions

The aim of the present study was to guide the enzymatic cocktail improvement to reduce the recalcitrant carbohydrate fraction characterizing the chemical composition of this material remaining after forced enzymatic hydrolysis of two pretreated lignocellulosic substrates (pretreated corn stover and pretreated sugarcane straw). The analysis was performed using techniques such as NMR or glycosyl residue composition by methylation analysis.

Recalcitrant material in both substrates represented approximately a 10 % of total carbohydrate left without being hydrolyzed. Two hypothesis were proposed to explain this gap: either the necessity of accessory enzymes to release a part of the polymer structure that was blocked by other modifications (such as acetyl/feruloyl resides), or an incomplete pretreatment of the biomass (i.e., due to the irregular particle size distribution or an inefficiently acid-exploded material attributable to high-scale operations), limiting the accessibility of the enzymes to the main polysaccharides of glucan and xylan.

Glycosyl composition analysis reveals that the recalcitrant material remains with glucose and xylose as the main monomers matching with the non-hydrolyzed material. Despite that some links could became slightly more represented, glycosyl linkage analysis reveals that 4-linked glucopyranosyl residue is the major component of all samples followed by a significant amount of 4-linked xylopyranosyl residues.

The results are consistent with the hypothesis that assigns the yield gap in hydrolysis to incomplete pretreatment leading to “pockets” of enzyme-inaccessible materials, instead to the need of alternative enzymes to process specific bonds linking the sugar polymers, because these links or polymers could not be found in the recalcitrant material. Besides according to Solid-state ^13^C nuclear magnetic resonance studies, this material has shown instead the presence of cellulose and hemicellulose and the chemical links corresponding to them.

This means that the hydrolytic yield limitations of the materials tested are likely caused by an incomplete pretreatment of the biomass and not in the enzyme cocktail preparations, which, on the other hand include all the enzymes required to hydrolyze the polysaccharides (mainly glucan and xylan) present in the recalcitrant material.

## Abbreviations

DMSO: dimethyl sulfoxide
DPMAS: direct polarization magic-angle spinning
EI-MS: electron impact ionization mode
GC/MS: gas chromatography/mass spectrometry
HPDC/MAS: high-power proton decoupling magic-angle spinning
HPLC: high-performance liquid chromatography
NMR: nuclear magnetic resonance spectroscopy
NREL: National Renewable Energy Laboratory
PCS: pretreated corn stover
PMAAs: partially methylated alditol acetates
PSCS: pretreated sugarcane straw
TFA: trifluoroacetic acid
TMS: per-*O*-trimethylsilyl
